# Depleting TMED3 alleviates the development of endometrial carcinoma

**DOI:** 10.1186/s12935-022-02649-0

**Published:** 2022-07-19

**Authors:** Jin Zhang, Yue Qi

**Affiliations:** Department of Obstetrics and Gynecology, Shengjing Hospital of China Medical University, No.36 Sanhao Street, Shenyang, Liaoning China

**Keywords:** Endometrial carcinoma, TMED3, Apoptosis, PI3K/AKT signaling

## Abstract

**Background:**

As one of gynecologic tumors, endometrial carcinoma (EC) has been characterized by high incidence rate, but its molecular pathogenesis has remained unclear. TMED3 is a membrane protein and has been indicated to implicate several tumor-related diseases. In the current study, we aimed to explore the physiological function of TMED3 in EC progression.

**Methods:**

Through bioinformatic analysis using The Cancer Genome Atlas database and immunohistochemistry assay on tissue microarray, we examined whether TMED3 was upregulated in EC tissues. After constructing TMED3-knockdown cell models via lentiviral transfection, qPCR and western blot were employed to determine the expression levels of TMED3 mRNA and protein. Then, Celigo cell counting assay, CCK8 assay, flow cytometry, wound-healing assay and Transwell assay were used to detect cell proliferation, cell cycle, cell apoptosis and cell migration, respectively.

**Results:**

As a result, it was found that TMED3 was upregulated in EC cells, which was also verified in clinical samples. We then found that downregulation of TMED3 considerably restrained cell cycle, cell growth and migration but promoted apoptosis of EC cells. The following *in-vivo* experiments also verified that tumor growth was inhibited after TMED3 knockdown. The exploration in molecular mechanisms showed that TMED3 deletion may weaken cellular viability through upregulating pro-apoptotic proteins and targeting PI3K/AKT signaling pathways.

**Conclusions:**

This study suggested that knocking down TMED3 affected the malignant phenotype of EC cells and thus limited tumor progression, which provided insights to the development of targeted drugs for EC treatment.

**Supplementary Information:**

The online version contains supplementary material available at 10.1186/s12935-022-02649-0.

## Background

Endometrial carcinoma (EC) is considered as the most common gynecologic tumor derived from the endometrium in developed nations and its incidence rate is gradually mounting worldwide [1]. Several factors are supposed to be the contributors to the prevalence of EC, such as ageing, obesity, diabetes and metabolic syndromes [2]. Most patients are diagnosed at the early stage and are able to receive effective and timely treatment as the clinical symptoms are quite evident [3]. Until now, the most common treatments of EC include surgery and adjuvant treatments such as radiotherapy and/or chemotherapy [4]. With the development in treatment for EC, the prognosis of patients has been greatly improved and the five-year survival rate is increasing [5]. However, those patients at advanced stage still have to suffer from poor prognosis and the deteriorating of this disease. A deeper understanding of the pathogenesis underlying EC development is of great help to discover novel therapeutic targets for EC patients.

*TMED3* (Transmembrane p24 trafficking protein 3) encodes a 217-aa long protein at 15q25.1 of human genome, which is involved in the transport and metabolism of proteins mediated by membrane-bounded vesicles. The proteins of p24 family are highly-conserved membrane proteins. These members form complexes and are responsible for the recognition of protein carriers and vesicle budding in the process of vesicular transport from endoplasmic reticulum to Golgi [6]. As a member of p24 protein family, TMED3 composes of a Golgi dynamics domain which enables to function in trafficking of intracellular proteins during Golgi dynamics [7]. TMED3 have been implicated to associated with several disorders. A previous study suggested that genetic mutations in a homolog of TMED3 in *Drosophila melanogaster*, Logjam, would upregulate some immune-regulated genes which were the downstream transcriptional targets of NF-κB or JNK pathways [8]. Moreover, Ha et al. [9] recently proposed that TMED3 was highly expressed in patients with clear cell renal cell carcinoma who suffered from poor prognosis, indicating that it may be a potential prognostic factor in the therapy of clear cell renal cell carcinoma. The oncogenic role of TMED3 was also verified in breast cancer development through clinicopathological data and *in vitro and vivo* experiments [10]. TMED3 knockdown also inhibited osteosarcoma cell proliferation and tumor growth by inhibiting RPS15A [11]. However, TMED3 was also reported as a metastatic suppressor and knocking down TMED3 could promote metastatic growth in human colon cancer [12]. Considering that controversial roles of TMED3 were reported in different cancers and little evidence was available in the context of EC, we here for the first time explored the biological role of TMED3 on EC progression.

Here, we would like to determine the biological functions of TMED3 and underlying molecular mechanisms in EC. We firstly investigated the expression of TMED3 using bioinformatic analysis and clinical tissue microarray, then tested cell proliferation, migration and apoptosis after establishing TMED3-knockdown cell models, as well as tumor growth in vivo. Our findings shed light upon the role of TMED3 played in EC progression and provided theoretical basis for targeted therapy against EC.

## Methods

### Bioinformatic analysis

We firstly analyzed the mRNA level of TMED3 derived from The Cancer Genome Atlas (TCGA) database. The RNA-sequencing was based on 547 EC samples and 35 normal tissues downloaded from the website (http://cancergenome.nih.gov/) and the RSEM (RNA-seq by expectation-maximization) values were used to represent the TMED3 expression level. T test was then employed to detect the difference in TMED3 expression between tumor and normal endometrium issues.

### Tissue microarray and immunohistochemistry (IHC)

EC tissues and para-carcinoma tissues were purchased from Shanghai Outdo Biotech Company (Cat. #HUteA060CS01). The tissue slides were baked in an oven at 65 °C for 30 min, dewaxed and then washed out with ethanol. After antigen retrieval in citric acid antigen at 100 °C for 10 min, all slides were blocked with 3% H_2_O_2_ for 5 min. Subsequently, TMED3 antibody (1:50, Cat. #ab151056, Abcam) was added at 37 °C for 1 h. After washing, the secondary antibody was added. Staining was accomplished using DAB and hematoxylin, then images were captured. Staining percentage scores were classified as: 1 (1–24%), 2 (25–49%), 3 (50–74%) and 4 (75–100%) and staining intensity were scored 0 (signless color) to 3 (light yellow, brown and dark brown). The IHC assay outcomes were determined by multiplying staining percentage and intensity scores.

### Lentiviral vector construction

Human gene *TMED3* was used as the template to design RNA interference target sequences (shTMED3-1, 5′-CTCTCACAAGACCGTCTACTT-3′, shTMED3-2, 5′-CACCTTCGAGCTGCCGGACAA-3′, shTMED3-3, 5′-CGTGAAGTTCTCCCTGGATTA-3′). cDNA containing interference sequence were connected to the linearized BR-V108 vector (Shanghai Yibeirui Biomedical Science and Technology Co., Ltd.) using restriction endonuclease Age I and EcoR I (Cat. #R3552L and R3101L, NEB). The junction products were transformed into *Escherichia coli* receptor cells (Cat. #CB104-03, TIANGEN) and positive clones were selected and verified by PCR. High-purity plasmids containing interference sequence were then extracted by EndoFree Maxi Plasmid Kit (Cat. #DP118-2, TIANGEN). Finally, 293T cells were co-transfected with BR-V108, Helper 1.0 and Helper 2.0 plasmids to obtain lentivirus.

### Cell lines and lentivirus transfection

The human EC cells, HEC-1-B cell line (Cat. #CL-0100, Shanghai Genechem Co., LTD.) and ishikawa cell line (Cat. #CL-0283, Shanghai Genechem Co., LTD.), were grown in 90% RPMI-1640 (Roswell park memorial institute-1640) (Cat. #10-040-CVB, Corning) containing 10% FBS (foetal bovine serum) (Cat. #16000-044, Invitrogen) and cultured in 90% DMEM (Dulbecco’s modified eagle medium) (Cat. #MCO-175, SANYO) containing 10% FBS. The culture incubator was moist air containing 5% CO_2_ at 37 °C. Cells were infected by lentivirus (shTMED3 or shCtrl) and cultured for 72 h infection efficiency was evaluated by observing the expression of green fluorescent protein (GFP) under a fluorescence microscope (Cat. #CKX31, OLYMPUS).

### Real-time quantitative polymerase chain reaction (qRT-PCR)

Total RNA was extracted from cell samples using Trizol (Cat. #T9424-100 m, Sigma), then RNA concentration and quality was detected by Nanodrop 2000/2000 C. cDNA for qRT-PCR was obtained by reverse transcription using Hiscript QRT supermix (+ gDNA WIPER) (Cat. #R123-01, Vazyme). PCR was performed by using AceQ qPCR SYBR Green Master Mix (Cat. #Q111-02, Vazyme). The 2^−∆∆CT^ method was applied for analyzing with GAPDH as the reference control. Primers used in PCR were as follows:

GAPDH, upstream, 5′-TGACTTCAACAGCGACACCCA-3′, downstream, 5′-CACCCTGTTGCTGTAGCCAAA-3′;

TMED3, upstream, 5′-GGCGTGAAGTTCTCCCTGGATT-3′, downstream 5’-GCTGTCGTACTGCTTCTTCGTTTC-3′.

### Western blotting (WB) assay

Protein expression levels were detected using WB. Cells were collected and lysed with 1×cold lysis buffer. Protein from cells was extracted with BCA protein detection kit (Cat. #23,225, HyClone-Pierce). Proteins were separated by 10% SDS-PAGE (sodium dodecyl sulfate polyacrylamide gel electrophoresis) and then transferred to a PVDF (Polyvinylidene fluoride) membrane and incubated with blocking liquid for 1 h. Detailed information on primary antibodies and secondary antibodies were presented in Additional file 1: Table S1. The membrane was color developed using ECL-PLUS/Kit (Cat. #RPN2232, Amersham).

### Cell proliferation assay

Proliferation of HEC-1-B and ishikawa cells was detected via Celigo cell counting assay and CCK8 assay. Regarding Celigo cell counting assay, the experimental cells were seeded in 96-well plates after lentivirus transfection and plates were scanned by Celigo Image Cytometer (Nexcelom) for consecutive five days at 10 am. The curve of the change multiple of the number of cells in the experimental group and the control group was then drawn. In CCK8 assay, we acquired cell suspension after digested by tyrisin which were then added into each well (2000 cells/well) of the 96-well plate (Cat. #3596, Cornning). Then we appended 10 µL CCK-8 (Cat. #96,992, Sigma) 2–4 h before termination of cell cultivation. Four hours later, we determined the absorption values at OD490 nm and finally got cell viability of the processed experimental cells.

### Flow Cytometry Assay (FACS)

Cell apoptosis and cell cycle of HEC-1-B and ishikawa cells was detected by flow cytometry. Cells were seeded and cultured for 5 days in 6 cm-well dishes. Afterwards, cells were digested with trypsin, centrifuged and washed with pre-cooled PBS (pH = 7.2 ~ 7.4). Then cells were re-suspended with 1×binding buffer. For apoptosis detection, cells were stained by Annexin V-APC for 15 min without light. Cell phase percentage was determined by FACSCalibur (BD Biosciences) to assess the apoptotic rate. For cell cycle detection, cell suspension was fixed with pre-cooled 70% ethanol (4 °C) for at least 1 h and then ethanol was removed. After centrifuged and washed, cells were stained PI staining solution (40×PI, 2 mg/mL: 100×RNase, 10 mg/mL: 1×PBS = 25:10:1000) for 30 min. FACSCalibur (BD Biosciences) was used to detect cell cycle.

### Wound-healing assay

HEC-1-B and ishikawa cells were seeded into 6-well dishes and grew for 72 h. Wounds crossing the cell layer were made by a 96-wounding replicator (VP scientific), and cell debris were tenderly washed. After cultured for 24 h, photographs were taken by fluorescence micrograph and the migration rate was calculated.

### Transwell assay

Lentiviral vector infected cells (5 × 10^4^ cell/well) were seeded into a 12-well cell culture insert which contains 8 μm polycarbonate membrane serum free medium. 30% FBS culture medium was added into the lower chamber. After 16 h culturing, cells migrating and adhering to the bottom of polycarbonate membrane were stained and pictures were obtained with a microscope (Olympus). We randomly three chambers and five fields of each chamber for counting the migrated cell numbers under the microscope. The migration rate was the percentage of the number of migrated cells in the shTMED3 group divided by the number of cells in the shCtrl group.

### Human apoptosis antibody array

Detection of related genes in human apoptosis signaling pathway was performed using Human Apoptosis Antibody Array (R&D Systems) following the manufacturer’s instructions. Briefly, ishikawa cells (shCtrl and shTMED3) were collected, washed and then lysed by lysis buffer. Total proteins were extracted and the concentrations were measured by BCA Protein Assay Kit (HyClone-Pierce). Each array antibody membrane was blocked, then incubated with protein samples (0.5 mg/mL) overnight at 4 °C and continuing incubated with HRP linked Streptavidin conjugate for 1 h. Enhanced chemiluminescence (ECL) (Amersham) was used for visualizing and the gray values were analyzed by ImageJ software. Specifically, the relative expression levels of apoptosis-related proteins were represented by each signal intensity (i.e., gray value) which was normalized by the positive control signal intensity.

### Mouse model

Four-week-old, female BALB/c nude mice (Beijing Vitalriver Experimental Animal Technology Co., Ltd) were randomly and evenly divided into shTMED3 and shCtrl groups. Mice xenograft models were established by subcutaneously injecting 0.2 mL (4 × 10^6^ cells/mL) stably-infected ishikawa cell suspensions. Mice weight, as well as tumor length and width were measured and recorded once a week. The tumor volume was calculated according to the following formula: π/6​×L×W×W, L represents the long diameter and W represents the short diameter. Forty-eight days post injection, all mice were anaesthetized by 0.7% sodium pentobarbital (10 µL/g, SIGMA) and *in-vivo* fluorescence expression detection was conducted by a Perkin Elmer IVIS Spectrum (Waltham). Next, the mice under anesthesia were killed by cervical dislocation and the tumors were removed for Ki-67 staining assay (Primary antibody: Ki-67, 1:200, Cat. #Ab16667, Abcam; Secondary antibody: 1:400, Cat. #ab6721, Abcam). All animal experiments performed in our study were approved by Ethics Committee of Shengjing Hospital of China Medical University.

### Statistics

All our cell experiments were performed in triplicate and the data were expressed as mean ± standard deviation (SD) and Student *t* test was used to compare the difference between two groups. Sign test was used to analyze the difference of TMED3 expression. Mann-Whitney *U* test and Spearman rank correlation analysis were applied to estimate the association between the expression pattern of TMED3 and clinical characteristics. Data processing was performed in SPSS 20.0 and plotting was proceeded in GraphPad Prism software 7.0. It was defined as significant when *P* was less than 0.05.

## Result

### Upregulation of TMED3 in EC tissues

**Fig. 1:**
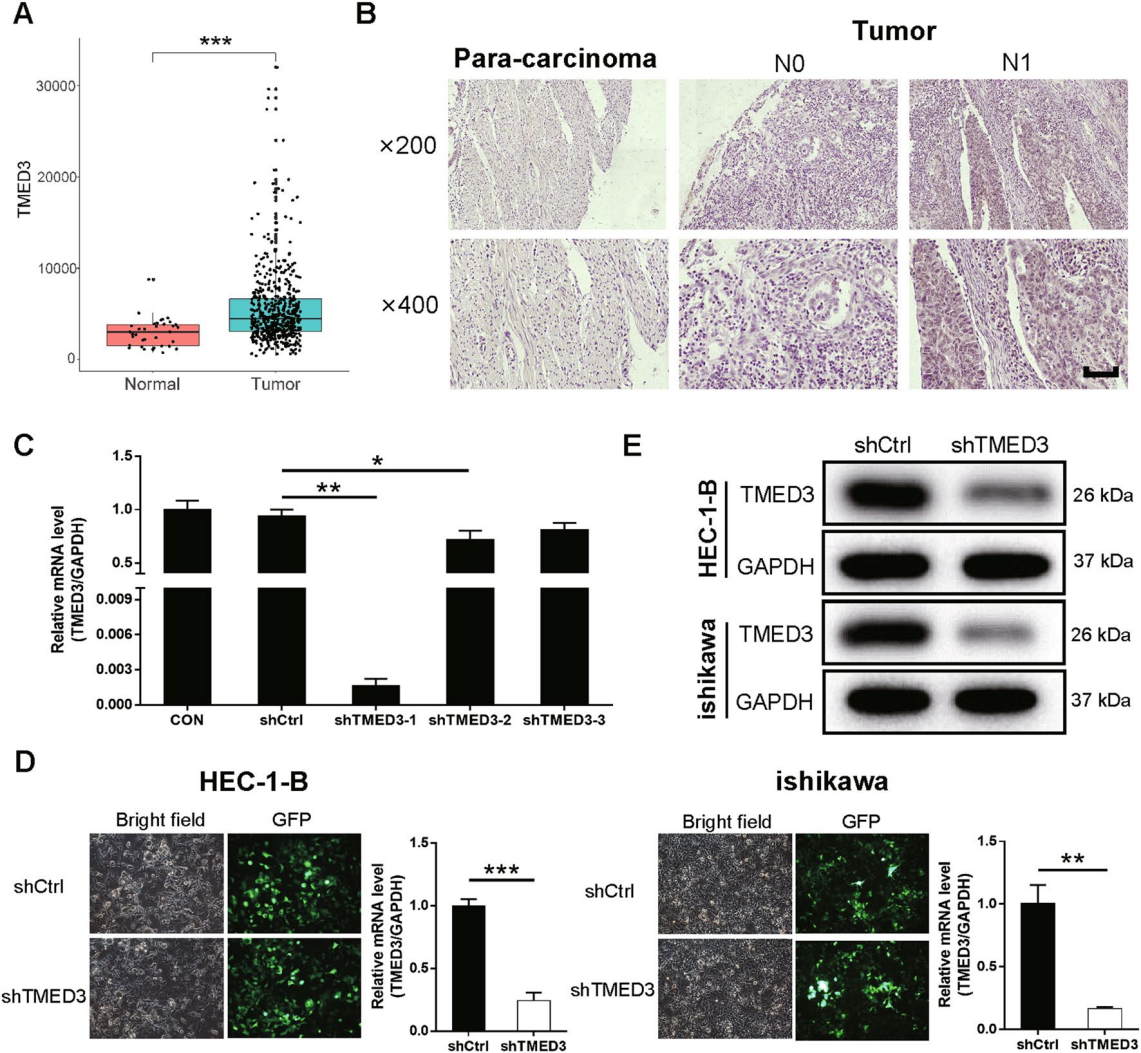
Overexpression of TMED3 in EC tissues and construction of TMED3-knockdown cell models. **A** Bioinformatic analysis derived from The Cancer Genome Atlas (TCGA) database revealed higher expression of TMED3 in EC tissues than that in normal endometrium issues. **B** The representative photos of para-carcinoma tissues and EC tissues deposed by IHC. **C** The knockdown efficiencies of three shTMED3-harboring lentiviruses in ishikawa cells. **D** The fluorescence images indicating high efficiency of lentivirus transfection (> 80%) and the relative mRNA levels of TMED3 in experimental HEC-1-B and ishikawa cells after treated by shCtrl-/shTMED3-harboring lentivirus detected by qRT-PCR. **E** The expression level of TMED3 protein in shCtrl-/shTMED3-harbroing cells of both cell lines. The scale bar is 50 μm long. *P < 0.05, **P < 0.01, ***P < 0.001

According to the bioinformatic analysis derived from TCGA database, the mRNA level of TMED3 were considerably higher in EC tumor tissues than that in normal endometrium tissues (*P* < 0.001, Fig. 1A). Through IHC assay on the tissue slides, we found that the expression of TMED3 was significantly higher in EC tissues than that in para-carcinoma tissues (*P* < 0.001, Table 1). We next detected whether TMED3 was significantly expressed based on the characteristics of patients or tumors, i.e., patient’s age, tumor size, tumor grade, the extent of tumor infiltration, tumor pathological stage, whether lymphatic metastasis occurred and whether tumor metastasis occurred. As a result, it was showed that whether the lymphatic metastasis occurred was predicted by the expression pattern of TMED3 (*P* = 0.038), but it was not the case regarding other patient’s/tumor’s characteristics (Table 2). The following Spearman correlation test also indicated that lymphatic metastasis of the tumor was positively correlated with the expression pattern of TMED3 (*r*_*s*_ = 0.385, *P* = 0.035, *N* = 30), which was also verified through IHC (Fig. 1B). Thus, we next constructed TMED3-knockdown cell models to test biological function of TMED3 in vitro and *in viv*o.

**Table 1:** Expression patterns of TMED3 in endometrial cancer (EC) tissues and para-carcinoma tissues obtained from immunohistochemistry analysis

TMED3 expression pattern	Tumor		Para-carcinoma		*P*
	No. of cases	Proportion	No. of cases	Proportion	
Low	19	57.6%	17	68.0%	< 0.001
High	14	42.4%	8	32.0%	

**Table 2:** Relationship between TMED3 expression patterns and characteristics of tumor or EC patients

Characteristics	No. of patients	TMED3 expression pattern		*P*
		Low	High	
All patients	33	19	14	
Age (years old)				
≤ 55^#^	17	10	7	0.883
> 55	16	9	7	
Tumor size (cm)				
< 3.5^#^	9	6	3	0.357
≥ 3.5	9	4	5	
Grade				
I	3	2	1	0.781
II	19	11	8	
III	9	5	4	
Tumor infiltration				
T1	18	11	7	0.543
T2	5	5	0	
T3	5	1	4	
Lymphatic metastasis (N)				
N0	22	15	7	0.038
N1	8	2	6	
Stage				
1	17	10	7	0.438
2	5	5	0	
3	9	3	6	
4	2	1	1	
Metastasis (M)				
M0	31	18	13	0.826
M1	2	1	1	

The label ^#^ indicates that 55 years old is the median age of the sampled patients and 3.5 cm is the median value of the sample tumors

### Construction of TMED3-knockdown cell models

After packaging RNAi lentivirus in ishikawa cells, the knockdown efficiencies were detected via qPCR and the results revealed that shTMED3-1 was the most effective shTMED3-harboring lentivirus and thus was selected for the following experiments (Fig. 1C). Subsequently, the selected shTMED3-harboring lentivirus and shCtrl-harboring lentivirus (negative control) were transfected into human EC cells (HEC-1-B and ishikawa cells). After verifying the efficiency of lentivirus transfection (> 80%) through fluorescence imaging, the relative mRNA level of TMED3 were examined using qRT-PCR and the results showed that TMED3 mRNA was significantly downregulated after knocking down TMED3 than that in controls in both HEC-1-B (*P* < 0.001) and ishikawa (*P* < 0.01) cells (Fig. 1D). WB assay also confirmed that TMED3 was dramatically downregulated in shTMED3 groups compared with that in shCtrl groups in both cell lines (Fig. 1E). In this case, the TMED3-knockdown cell models were successfully established.

### Limitation in tumor cell growth and migration induced by depleting TMED3

**Fig. 2:**
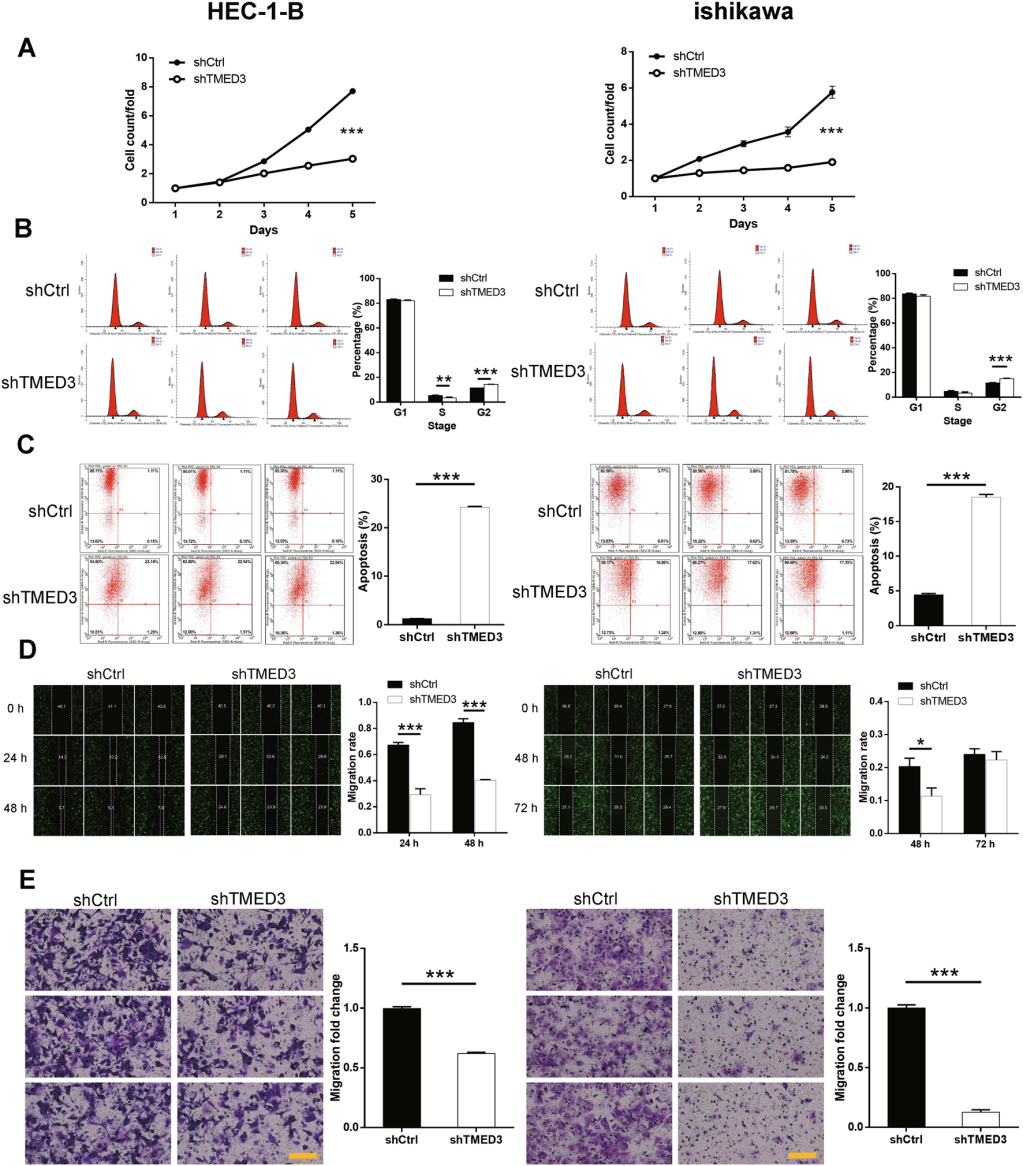
TMED3 depletion restrains cell proliferation and migration but promotes cell apoptosis. **A** The comparison in cell proliferation of the experimental groups (shCtrl vs. shTMED3) of HEC-1-B and ishikawa cell lines resulted by Celigo cell counting assay. **B** The cell cycle detected by FACS and the comparison in cell percentages of three cell cycle stages in the experimental groups (shCtrl vs. shTMED3) of both cell lines. **C** The difference in the percentage of cell apoptosis in the cell groups (shCtrl vs. shTMED3) of both cell lines. The red-R-fluorescence on the x-axis in the images represented the percentage of apoptotic cells. The y-axis being marked Green-B fluorescence represented the fluorescence signal of the GFP label on the lentivirus used to transfect HEC-1-B and ishikawa cells, which ensured that the detected cells were all successfully transfected cells. **D** The changes in cell migration ability after scratching the wound in the experimental groups (shCtrl vs. shTMED3) of both cell lines. **E** The representative photographs (×200) and the comparison in cell migration derived from Transwell assay in cell groups (shCtrl vs. shTMED3) of both cell lines. The left and right panels are experimental results for HEC-1-B cells and ishikawa cells, respectively. The scale bar is 100 μm long. *P < 0.05, **P < 0.01, ***P < 0.001

The results of Celigo cell counting assay revealed that cell growth was substantially restricted after transfection of shTMED3-harboring lentivirus in both HEC-1-B (*P* < 0.001) and ishikawa (*P* < 0.01) cells (Fig. 2A). The findings from FACS showed that the amounts of cells in G1 stage did not differ between shCtrl and shTMED3 groups in two cell lines. HEC-1-B cells in S stage in shTMED3 group was significantly less than that in shCtrl group (*P* < 0.01), while no difference was detected in ishikawa cells. However, cells in G2 stage accounted more in shTMED3 groups than controls in both HEC-1-B and ishikawa cell lines (*P* < 0.001) (Fig. 2B). As expected, the percentages of apoptotic cells were much larger in shTMED3-harboring cells than those in control groups in both cell lines (HEC-1-B, *P* < 0.001; ishikawa, *P* < 0.001) (Fig. 2C). In regarding to the detection of cell migration, wound-healing assay indicated the migration ability of HEC-1-B cells considerably weakened followed by TMED3 knockdown in comparison with controls 24 h (*P* < 0.001) and 48 h (*P* < 0.001) after scratching the wound, while ishikawa cells in shTMED3 group migrated a shorter distance than controls 48 h after the scratching only (*P* < 0.05) (Fig. 2D). The results of Transwell assay also suggested that knocking down TMED3 substantially limited cell migratory ability in the experimental cell lines (HEC-1-B, *P* < 0.001; ishikawa, *P* < 0.001) (Fig. 2E). In summary, the aforementioned *in-vitro* experiments demonstrated that depletion of TMED3 in EC cells could restrain cell proliferation and cell migration, but promote cell apoptosis.

### Underlying molecular mechanism of TMED3 in EC cells

**Fig. 3:**
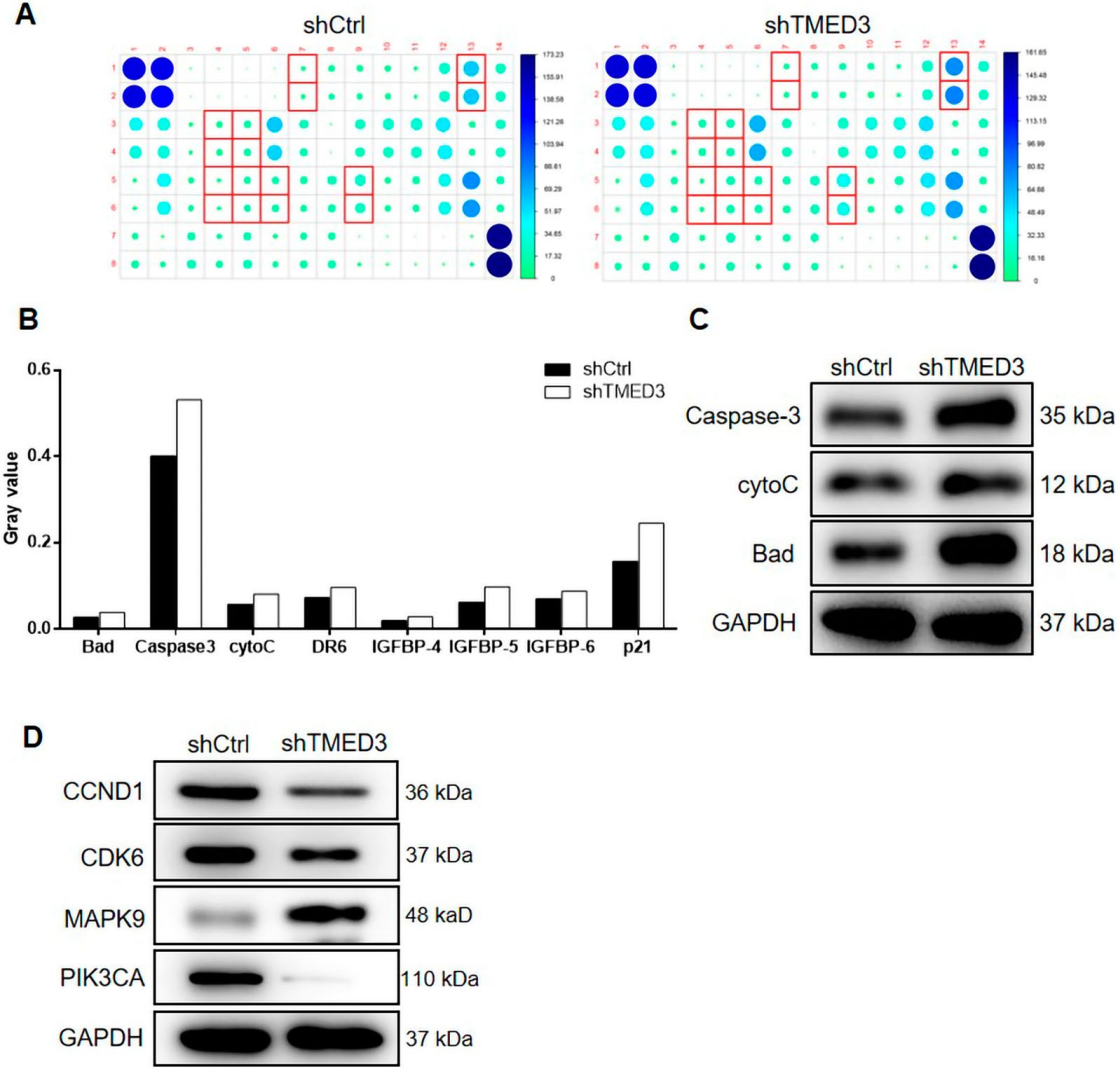
Downstream molecular mechanism of TMED3 in ishikawa cells. **A**-**B** The comparison in expression levels of some apoptosis-related proteins mediated by TMED3 in the cell models (shCtrl vs. shTMED3). Each column is the mean value of the two gray values in the Human apoptosis antibody array. **C** Changes in some apoptosis-related proteins mediated by TMED3 detected by WB assay. **D** Changes in some TMED3-mediated proteins involved in PI3K/AKT and MAPK signaling pathways

In light of the findings above, we detected the expression levels of apoptosis-related proteins in shCtrl and shTMED3 groups of ishikawa cells. We found that there were eight proteins upregulated in the shTMED3-harboring cells compared with those in controls, i.e., Bad, Caspase3, cytoC, DR6, IGFBP-4, IGFBP-5, IGFBP-6 and p21 (Fig. 3A–B). WB assay also revealed that Bad, Caspase-3 and cytoC were significantly upregulated following TMED3 knockdown in ishikawa cells (Fig. 3C). Regarding cell cycle-related proteins, we found that CCND1, CDK6 and PIK3CA were downexpressed while MAPK9 was upregulated when TMED3 was silenced by lentivirus in comparison with those in shCtrl group of ishikawa cells (Fig. 3D), indicating PI3K/AKT pathway may be one of the possible downstream regulatory pathways mediated by TMED3 in EC cells. In this case, we further performed rescue experiments and examined changes in cell phenotype following PI3K inhibitor (LY294002, Cat. #S1105, Selleck) treatment.

**Fig. 4:**
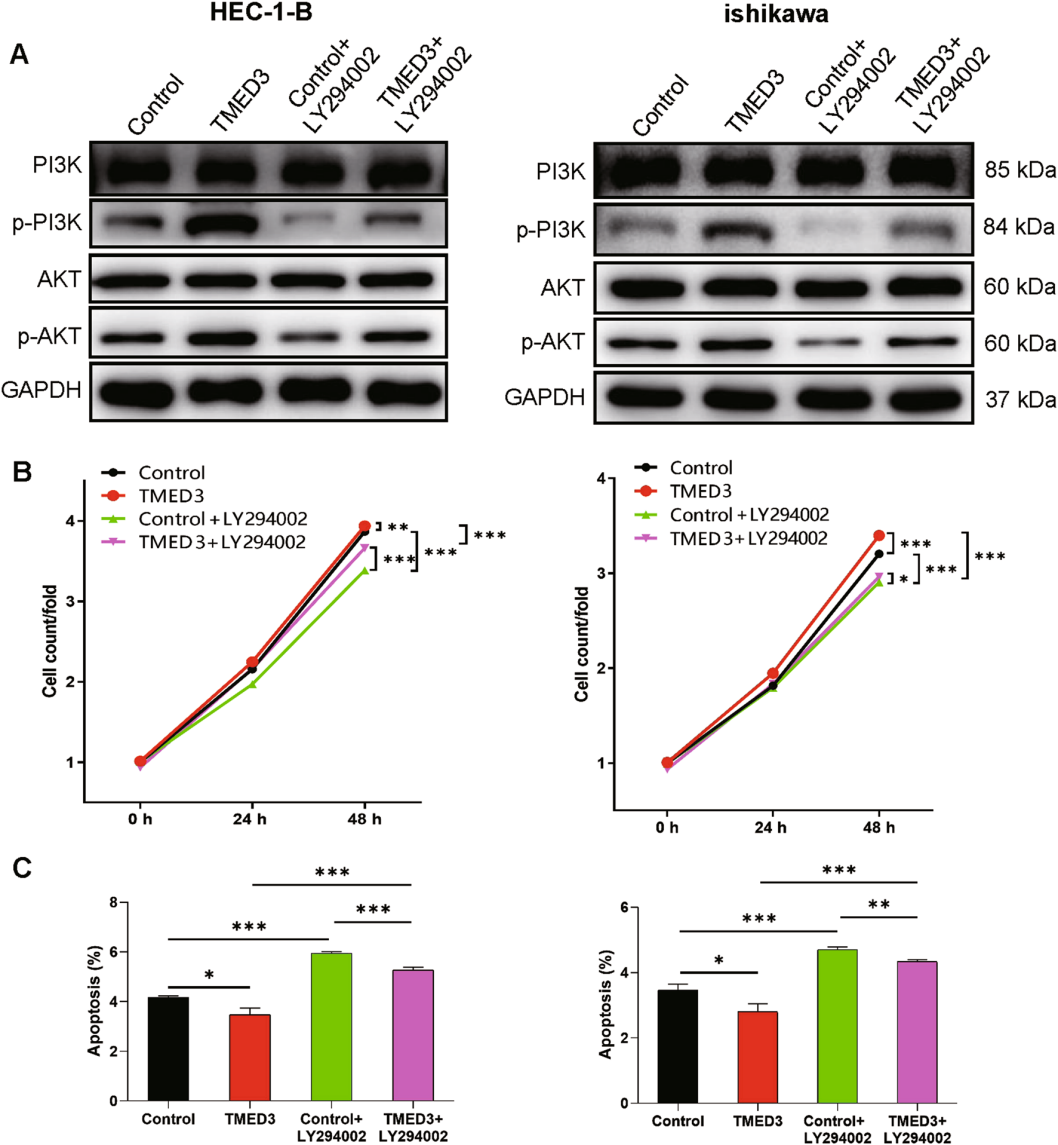
TMED3 mediated EC cell phenotype via PI3K/AKT signaling pathway. **A** The expression levels of PI3K, p-PI3K, AKT and p-AKT in HEC-1-B and ishikawa cells in each cell group. **B** The comparisons in cellular ability of proliferation among four cell groups. **C** The comparisons in percentages of apoptotic cells among four cell groups. *P < 0.05, **P < 0.01, ***P < 0.001

Specifically, we established four cell groups, Control group (stably-transfected by empty plasmid), TMED3 group (TMED3 overexpression), Control + LY294002 (stably-transfected by empty plasmid and treated by PI3K inhibitor) and TMED3 + LY294002 (TMED3 overexpression and treated by PI3K inhibitor). As a result, phosphorylation levels of PI3K at Tyrosine 607 and AKT at Serine 473 were considerably restrained following the treatment of PI3K inhibitor in HEC-1-B and Ishikawa cells, as suggested by WB assay in Fig. 4A. Overexpression of TMED3 promoted the cellular ability to proliferate, as showed by CCK8 assay (Fig. 4B), and PI3K inhibitor could significantly reverse the promoting effects resulted by TMED3 upregulation (HEC-1-B cells, *P* < 0.001; Ishikawa cells, *P* < 0.001). With regard to cell apoptosis determined by FACS, we found that PI3K inhibitor substantially increased the percentage of apoptotic cells despite overexpression of TMED3 in both cell lines (HEC-1-B cells, *P* < 0.001; Ishikawa cells, *P* < 0.001) (Fig. 4C). As a result, we suggested that TMED3 mediated cell phenotype and functioned as a tumor promoter in EC cells via targeting PI3K/AKT signaling pathway.

### Restrictions on tumor growth *in vivo* after TMED3 knockdown

**Fig. 5:**
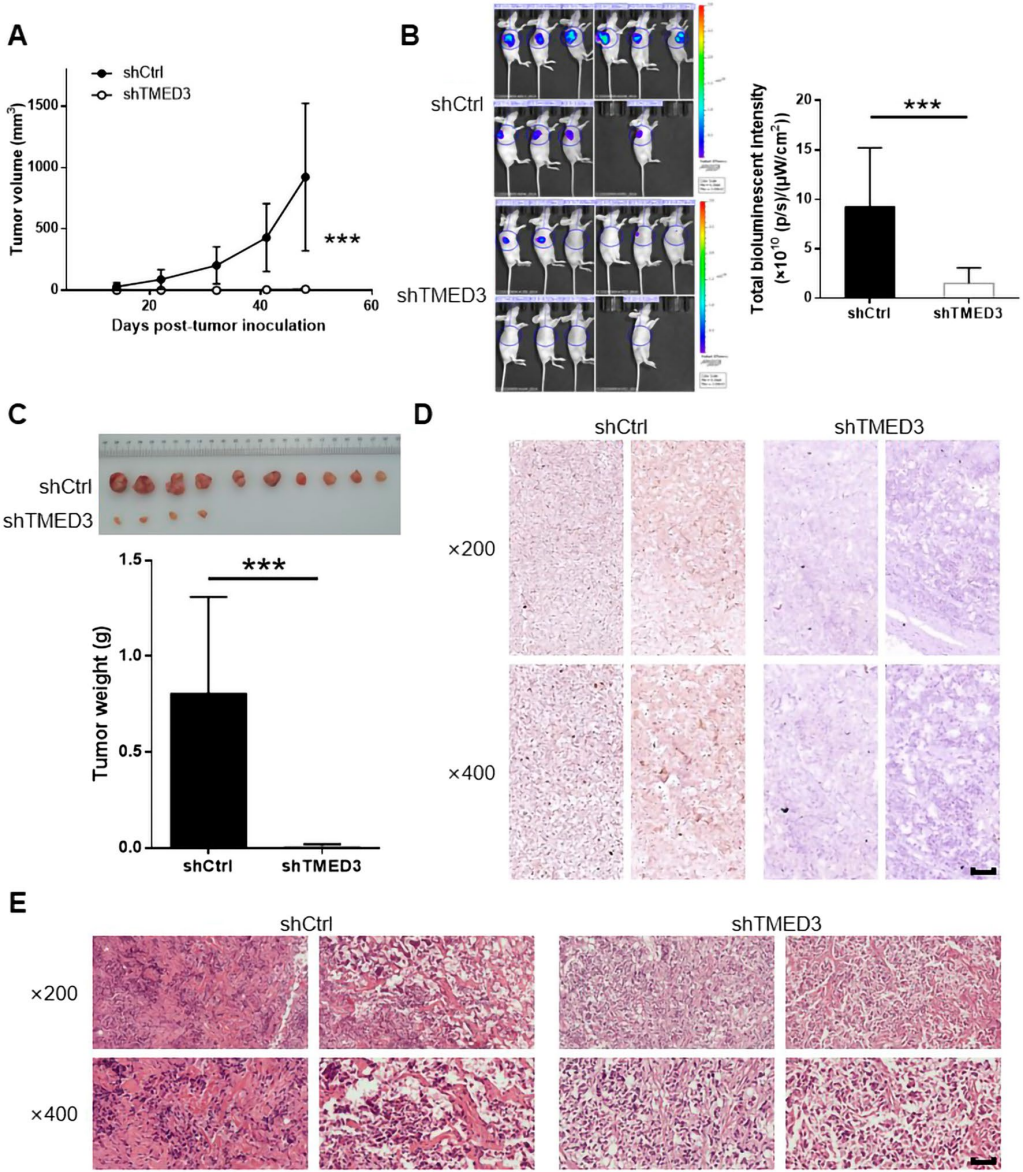
TMED3 knockdown limits tumor growth *in vivo*. **A** The comparison in tumor volume between mouse models of shCtrl and shTMED3 groups after injected ishikawa cells. **B** The bioluminescence images of the experimental mice and the comparison in the total bioluminescence intensity of mouse models (shCtrl vs. shTMED3). **C** The photo of removed solid tumors from experimental mice and the difference in the tumor weight between shCtrl and shTMED3 groups. **D** Pathological images of the solid tumors after TMED3 staining in shCtrl and shTMED3 groups. **E** Pathological images of the solid tumors after Ki-67 staining in shCtrl and shTMED3 groups. The scale bar is 50 μm long. ***P < 0.001

To verify the role TMED3 played in vivo, the mice xenograft models were constructed and we found the solid tumors in mice of shCtrl group grew much faster and larger than those in shTMED3 group (*P* < 0.001) (Fig. 5A). The total bioluminescence intensity in the experimental animals of shTMED3 group were much less than controls, either (*P* < 0.001) (Fig. 5B). We also found that the solid tumors in shCtrl group were much heavier than those in shTMED3 group after removing them from mice (*P* < 0.001) (Fig. 5C). Besides, the expression of TMED3 in shTMED3 group was inhibited (Fig. 5D). Lastly, Ki-67 staining revealed that, compared with that in shTMED3 group, removed tumors from shCtrl group exhibited upregulation of Ki-67 (Fig. 5E). These results suggested that TMED3 may be a promoter to EC tumor growth.

## Discussion

In recent years, the incidence of EC continues to rise among younger obese women [13] and surgical resection is considered as the first-line therapy. In view of the side effects of the following radiotherapy and/or chemotherapy, targeted therapy is suggested to be a better way for EC treatment. In the current study, we detected the expression of TMED3, which played a critical role in vesicular protein trafficking at ER-Golgi interface in eukaryotic cells [6] in clinical specimens and EC cell lines. Our findings suggested that TMED3 was highly expressed in clinical samples and could predict the lymphatic metastasis in EC patients. Silencing TMED3 in human EC cells using lentiviral transfection significantly inhibited cell viability and migration, as well as cell cycle progression but increased the amount of apoptotic cells. Also, TMED3 knockdown imposed restrictions on tumor growth in the mouse xenograft models. Evidence from in vitro and in vivo experiments demonstrated the tumor-promoting effects of TMED3 in EC development.

Consistently, in patients with chordoma, which was considered as a low-grade tumor derived from axial skeleton, TMED3 was also found overexpression in tumor cells [14]. In addition, depletion of TMED3 was associated with the increase of cell apoptosis and the decrease of cell growth and migration. In breast cancer cells, the elevated expression level of TMED3 was significantly correlated with the level of estrogen receptor, progesterone receptor and human epidermal growth factor receptor-2, as well as the status of lymph nodes metastasis [15]. In consistence with our study, knocking down TMED3 in breast cancer cell models dramatically limited cell colony formation and cell motility by Wnt/β-catenin signaling [16]. It has been suggested that TMED3 is involved in chemotherapy resistance during the treatment for gastric cancer. Specifically speaking, TMED3 siRNA enabled tumor cells to increase cisplatin sensitivity [17]. In regarding to the downstream of TMED3 in tumor, a previous study proposed that TMED3 promoted metastasis of hepatocellular carcinoma and served as a contributor in tumor progression via IL-11/STAT3 signaling pathway [18]. Besides, it was proposed that TMED3 enhanced Wnt/β-catenin signaling by regulating AKT to exert a tumor-promoting function in non-small cell lung cancer [19].

We also performed the preliminary exploration in downstream regulatory pathways of TMED3 in EC cells and indicated that TMED3 depletion downregulated CCND1, CDK6 and PIK3CA but upregulated MAPK9. As is known, CCND1 could form a cell cycle-dependent complex with CDK6 as a regulatory subunit which is indispensable in G1/S transition [20], which may cause the block of cell mitosis in S stage following TMED3 depletion in EC cells. Moreover, it is well known that PI3K/AKT pathway is closely linked with tumorigenesis and progression in various tumors, e.g., breast and gynecologic malignancies [21], hepatocellular carcinoma [22]. As an important subunit of PI3K, PIK3CA acts as a critical role in activating the PI3K/AKT signaling pathway [23], which is proved to be regulated by TMED3. Furthermore, MAPK9 upregulation mediated by TMED3 depletion was determined in our study. As a member of MAPK protein family, MAPK9 plays a part in multiples signals of mammalian cells [24] and is found improperly activated or inactivated in cells of different cancer types. For example, MAPK9 is a contributor to the development and progression of glioma, prostate carcinoma or lung carcinoma [25], but acts as a negative regulator in cellular proliferation [26]. Our findings verified the negatively regulatory function of MAPK9 mediated by TMED3 in EC cells. Besides, the apoptosis-related proteins regulated by TMED3 varied in different tumor cells. In chordoma cells, the anti-apoptotic protein Bcl-2 was upregulated in shTMED3 cell group [14]. However, we here detected overexpression of several pro-apoptotic proteins, such as Caspase3, which was responsible for the execution phase during cell apoptosis. Nonetheless, there are some limitations in this study. For example, we need to expand the number of EC tissues samples to verify the high expression of TMED3 in EC. Second, we need to further verify which apoptotic-related protein specifically TMED3 plays a role in EC cell function.

## Conclusions

In a word, this study demonstrated overexpression of TMED3 in endometrial carcinoma cells and TMED3 depletion significantly inhibited tumor progression both in cell and animal models through limiting growth and migration but promoting apoptosis of EC cells. Apoptosis-related proteins mediated by TMED3 included Bad, Caspase3, cytoC, DR6, IGFBP-4, IGFBP-5, IGFBP-6 and p21, which were upregulated following TMED2 knockdown. Moreover, some key proteins involved in PI3K/AKT and MAPK signaling pathways were dysregulated, i.e., CCND1, CDK6, PIK3CA and MAPK9, in the shTMED3-harboring cell group. We suggested that TMED3 functioned as a tumor promoter and played an important role in EC progression via regulating PI3K/AKT signaling pathway. These findings indicated the contributing role of TMED3 in the development of EC which may serve as a therapeutic target for EC treatment.

## Supplementary Information

**Additional file 1: Table S1** Antibodies involved in western blotting-based assay in this study.

## Data Availability

All data generated or analysed during this study are included in this article.

## References

[1] Morice P, Leary A, Creutzberg C, Abu-Rustum N, Darai E (2016). Endometrial cancer. Lancet.

[2] Trabert B, Wentzensen N, Felix AS, Yang HP, Sherman ME, Brinton LA (2015). Metabolic syndrome and risk of endometrial cancer in the united states: a study in the SEER-medicare linked database. Cancer Epidemiol Biomarkers Prev.

[3] Brooks RA, Fleming GF, Lastra RR, Lee NK, Moroney JW, Son CH (2019). Current recommendations and recent progress in endometrial cancer. CA Cancer J Clin.

[4] Sorosky JI (2012). Endometrial cancer. Obstetrics Gynecol.

[5] Liu Y, Chang Y, Cai Y (2020). Hsa_circ_0061140 promotes endometrial carcinoma progression via regulating miR-149–5p/STAT3. Gene.

[6] Jerome-Majewska LA, Achkar T, Luo L, Lupu F, Lacy E (2010). The trafficking protein Tmed2/p24beta(1) is required for morphogenesis of the mouse embryo and placenta. Dev Biol.

[7] Jenne N, Frey K, Brugger B, Wieland FT (2002). Oligomeric state and stoichiometry of p24 proteins in the early secretory pathway. J Biol Chem.

[8] Boltz KA, Carney GE (2008). Loss of p24 function in Drosophila melanogaster causes a stress response and increased levels of NF-kappaB-regulated gene products. BMC Genomics.

[9] Ha M, Moon H, Choi D, Kang W, Kim JH, Lee KJ (2019). Prognostic role of TMED3 in clear cell renal cell carcinoma: a retrospective multi-cohort analysis. Front Genet.

[10] Pei J, Zhang J, Yang X, Wu Z, Sun C, Wang Z (2019). TMED3 promotes cell proliferation and motility in breast cancer and is negatively modulated by miR-188-3p. Cancer Cell Int.

[11] Xu W, Li Y, Ye X, Ji Y, Chen Y, Zhang X (2021). TMED3/RPS15A Axis promotes the development and progression of osteosarcoma. Cancer Cell Int.

[12] Duquet A, Melotti A, Mishra S, Malerba M, Seth C, Conod A (2014). A novel genome-wide in vivo screen for metastatic suppressors in human colon cancer identifies the positive WNT-TCF pathway modulators TMED3 and SOX12. EMBO Mol Med.

[13] Lu KH, Broaddus RR (2020). Endometrial cancer. N Engl J Med.

[14] Yang J, Huang H, Xiao D, Duan Y, Zheng Y, Chen Z (2021). Knockdown of TMED3 inhibits cell viability and migration and increases apoptosis in human chordoma cells. Int J Oncol.

[15] Pei J, Zhang J, Yang X, Wu Z, Sun C, Wang Z (2019). TMED3 promotes cell proliferation and motility in breast cancer and is negatively modulated by miR-188–3p. Cancer Cell Int.

[16] Zhang X, Luo Y, Li Q (2020). TMED3 promotes proliferation and migration in breast cancer cells by activating Wnt/beta-Catenin signaling. Onco Targets Ther.

[17] Peng C, Huang K, Liu G, Li Y, Yu C (2019). MiR-876-3p regulates cisplatin resistance and stem cell-like properties of gastric cancer cells by targeting TMED3. J Gastroenterol Hepatol.

[18] Zheng H, Yang Y, Han J, Jiang W-H, Chen C, Wang M-C (2016). TMED3 promotes hepatocellular carcinoma progression via IL-11/STAT3 signaling. Sci Rep.

[19] Zhang D, Sun L, Zhang J (2021). TMED3 exerts a protumor function in non-small cell lung cancer by enhancing the Wnt/beta-catenin pathway via regulation of AKT. Toxicol Appl Pharmacol..

[20] Li N, Zeng J, Sun F, Tong X, Meng G, Wu C (2018). p27 inhibits CDK6/CCND1 complex formation resulting in cell cycle arrest and inhibition of cell proliferation. Cell Cycle.

[21] Janku F, Wheler JJ, Westin SN, Moulder SL, Naing A, Tsimberidou AM (2012). PI3K/AKT/mTOR inhibitors in patients with breast and gynecologic malignancies harboring PIK3CA mutations. J Clin Oncol: official journal of the American Society of Clinical Oncology.

[22] Chai R, Fu H, Zheng Z, Liu T, Ji S, Li G (2017). Resveratrol inhibits proliferation and migration through SIRT1 mediated post-translational modification of PI3K/AKT signaling in hepatocellular carcinoma cells. Mol Med Rep.

[23] Huang T, Ren K, Ding G, Yang L, Wen Y, Peng B (2020). miR-10a increases the cisplatin resistance of lung adenocarcinoma circulating tumor cells via targeting PIK3CA in the PI3K/Akt pathway. Oncol Rep.

[24] Yoon CH, Kim MJ, Kim RK, Lim EJ, Choi KS, An S (2012). c-Jun N-terminal kinase has a pivotal role in the maintenance of self-renewal and tumorigenicity in glioma stem-like cells. Oncogene.

[25] Bubici C, Papa S (2014). JNK signalling in cancer: in need of new, smarter therapeutic targets. Br J Pharmacol.

[26] Sabapathy K, Wagner EF (2004). JNK2: a negative regulator of cellular proliferation. Cell Cycle.

