# Supplementary material for: Depleting TMED3 alleviates the development of endometrial carcinoma
Source: Cancer Cell Int. 2022 Jul 19;22:231. doi: 10.1186/s12935-022-02649-0 (PMC9295347; doi:10.1186/s12935-022-02649-0)
Supplement: Supplementary file 1 — Additional file 1: Table S1 Antibodies involved in western blotting-based assay in this study. [file 12935_2022_2649_MOESM1_ESM.docx]

**Table S1** Antibodies involved in western blotting-based assay in this study.

|  | Primary antibody | Size/kDa | Diluted multiples | Source | Company | Serial number |
| --- | --- | --- | --- | --- | --- | --- |
| Figure 1E | TMED3 | 26 | 1:1000 | Rabbit | abcam | 21902-1-AP |
|  | GAPDH | 37 | 1:3000 | Rabbit | Bioworld | AP0063 |
| Figure 3C | Caspase-3 | 35 | 1:1000 | Rabbit | CST | 9662S |
|  | cytoC | 12/15 | 1:2000 | Mouse | Abcam | ab13575 |
|  | Bad | 18/23 | 1:2000 | Rabbit | Abcam | ab32445 |
|  | GAPDH | 36 | 1:30000 | Mouse | Proteintech | 60004-1-lg |
| Figure 3D | CCND1 | 36 | 1:2000 | Rabbit | Abcam | ab134175 |
|  | CDK6 | 37 | 1:1000 | Rabbit | Abcam | ab151247 |
|  | MAPK9 | 48 | 1:3000 | Rabbit | Abcam | ab76125 |
|  | PIK3CA | 110 | 1:1000 | Rabbit | Abcam | ab40776 |
|  | GAPDH | 37 | 1:3000 | Rabbit | Bioworld | AP0063 |
| Figure 4A | PI3K | 85 | 1:1000 | Rabbit | CST | 4257S |
|  | p-PI3K | 84 | 1:500 | Rabbit | Abcam | ab182651 |
|  | AKT | 60 | 1:3000 | Mouse | Proteintech | 66444-1-Ig |
|  | p-AKT | 60 | 1:2000 | Rabbit | CST | 4060S |
|  | GAPDH | 36 | 1:30000 | Mouse | Proteintech | 60004-1-lg |

| Secondary antibody | Diluted multiples | Company | Serial number |
| --- | --- | --- | --- |
| Goat Anti-Rabbit | 1:3000 | Beyotime | A0208 |
| Goat Anti-Mouse | 1:3000 | Beyotime | A0216 |
